# Bacterial inoculations can perturb the growth trajectory of diatoms with an existing microbiome

**DOI:** 10.7717/peerj.8352

**Published:** 2020-01-27

**Authors:** Lydia J. Baker, Paul F. Kemp

**Affiliations:** 1 Microbiology, Cornell University, Ithaca, NY, United States of America; 2 Oceanography Department, University of Hawai’i Mānoa, Honolulu, HI, United States of America

**Keywords:** Algal-bacterial interaction, Symbiosis, Microbiome, Xenic, Marine diatom

## Abstract

Inoculation of axenic diatom monocultures with individual bacterial strains has been used effectively to examine the relationship between bacteria and a diatom host. Both beneficial and harmful effects on diatom fitness have been observed. Yet, diatoms commonly host a consortium of bacteria that could influence their response to perturbation by bacterial inoculations. In this study, diatom cultures with an existing microbiome were inoculated with individual bacterial strains. Strains of two genera of bacteria commonly found associated with diatoms (*Alteromonas* and *Marinobacter*) were isolated from a culture of the diatom *Chaetocero*s sp. KBDT20. To evaluate whether bacterial inoculations can impact the growth, peak abundance, or decline of diatoms with an intact microbiome, individual bacterial strains were inoculated into batch cultures of the original host as well as two non-origin diatom hosts (*Chaetoceros* sp. KBDT32 and *Amphiprora* sp. KBDT35). Inoculations were repeated under vitamin-replete and vitamin-deficient conditions to assess whether vitamin concentration modulates the impact of bacterial inoculations on the host. The origin *Chaetoceros* culture was largely unperturbed by bacterial inoculations. In contrast, non-origin hosts experienced long-term impacts on their growth trajectory, and those impacts were found to be dependent on the concentration of vitamins in the growth medium. For the non-origin *Chaetoceros*, all positive impacts were observed in vitamin-replete conditions and all negative impacts were observed in vitamin-deficient conditions. *Amphiprora* was only impacted by inoculation with *Marinobacter* strains in vitamin-deficient conditions, and the effect was negative. Neither individual bacterial strains nor genera resulted in exclusively beneficial nor detrimental impacts, and the magnitude of effect varied among bacterial strains. This study demonstrates that bacterial inoculations can have long-lasting impacts on the growth trajectory of diatoms with an existing microbiome, that this impact can differ even between congeneric diatoms, and that the impact can be significantly modulated by vitamin concentration.

## Introduction

For nearly 200 million years diatoms and bacteria have coexisted in the marine environment, and their interactions likely influenced their ecological successes and failures (Amin, Parker & Armbrust, 2012). Diatoms are the base of the food web in many ecosystems and export a significant amount of carbon from the photic zone, even in oligotrophic systems where they are relatively uncommon (Michaels & Silver, 1988; Brzezinski et al., 2011; Karl et al., 2012). Bacteria can stimulate diatom host growth, affect bloom duration, and induce diatom aggregation (Smith et al., 1995; Gärdes et al., 2011; Amin et al., 2015); therefore, understanding the influence of bacteria is vital to understanding the ecology of diatoms.

Diatom-bacterial interactions can be beneficial or harmful to either member of the association. At all stages of growth diatoms leak organic substances that are primarily composed of carbohydrates (80–90%) (Myklestad, 1995), and attract and retain bacteria (Kogure, Simidu & Taga, 1982; Barbara & Mitchell, 2003). Bacteria attached to a diatom thus benefit from having access to a reliable source of organic nutrients (Azam, Fenchel & Field, 1983). Interactions between diatoms and surface-associated bacteria can be commensal, wherein bacteria benefit from being associated with diatoms, but diatoms receive neither benefit nor harm (Rosowski, 1992; Droop, 2007). Bacteria can also act to the detriment of a diatom host, and in turn, diatoms can deter harmful bacteria by producing antimicrobial phlorotannins, fatty acids, polysaccharides, peptides, and terpenes (Shannon & Abu-Ghannam, 2016). At least some diatoms may benefit from interactions with bacteria, and may, in fact be dependent on interactions with bacteria that provide limiting nutrients. For example, all species within the *Chaetoceros* genus are thought to be vitamin B_12_ auxotrophic (King et al., 2011), therefore, harboring vitamin B_12_-producing bacteria may benefit the diatom host cell (Croft et al., 2005). Bacteria associated with diatoms may also stimulate host cell division, prevent virus-induced host cell lysis, protect against algicidal bacteria, produce micronutrients, or supply biologically available iron (Croft et al., 2005; Amin et al., 2009; Amin et al., 2015; Amin, Parker & Armbrust, 2012; Kimura & Tomaru, 2014).

The most common approach used to examine diatom-bacteria interactions utilizes axenic diatom cultures that are perturbed by the introduction of a single bacterial strain. Numerous studies have shown that adding individual bacterial strains to an axenic host can profoundly impact the host’s growth (Grossart, 1999; Gärdes et al., 2012; Sison-Mangus et al., 2014; Durham et al., 2015; Zecher et al., 2015; Han et al., 2016). The simplified model system of axenic host and bacteria monoculture is useful to study host-bacterial interactions, but such simplified populations are atypical of diatoms in nature.

Diatoms in nature are commonly found with a consortium of associated bacteria (Amin, Parker & Armbrust, 2012; Baker & Kemp, 2014), and when deprived of their resident bacteria some diatoms grow poorly or not at all (Windler et al., 2014; Sison-Mangus et al., 2014; Amin et al., 2015). Diatoms can host multiple genera of bacteria on a single cell (Baker & Kemp, 2014), thus multiple host-bacteria and bacteria-bacteria interactions may be occurring simultaneously. During previous studies of the composition of bacteria attached to *Chaetoceros* cells, bacterial interactions were found to influence the composition of diatom-attached bacteria (Baker, Alegado & Kemp, 2016), potentially affecting the host indirectly. The host cell has the potential to be impacted by the bacterial inoculum directly through interactions of inoculated bacteria with the host, or indirectly through interactions of inoculated bacteria with the existing bacterial microbiome co-cultured with the host. Interactions between the inoculum and co-cultured bacteria may occur through processes such as bacterial production of antibiotics, co-metabolism, or competition, all of which may secondarily influence the interactions of host-associated bacteria. Additionally, these interactions are not static, and can be modulated by the host growth state, interactions with other bacteria, and nutrient concentrations (Kogure, Simidu & Taga, 1982; Grossart et al., 2005; Baker, Alegado & Kemp, 2016).

The present study takes the unique approach of examining how additions of single bacterial strains impact a diatom host with an existing microbiome. The existing microbiome is not intended to be representative of any particular location, season, or ecological environment; it is the microbiome co-cultured with the original single-cell diatom isolate. However, the diatom microbiome may be maintained, even in laboratory conditions (Behringer et al., 2018). Using hosts with intact microbiomes, our experimental design addresses nearly the same question as previous studies using axenic-diatom model systems: does the addition of single bacterial strains impact the growth trajectory of *xenic* hosts?

In the present study, the impact of bacterial inoculation on a host is measured through observed differences in growth, maximum abundance, or decline in comparison to a control. Three different diatom hosts were tested: a centric diatom from which the bacterial strains used as inocula in this study were cultured (the origin host *Chaetoceros* sp. KBDT20; Baker, Alegado & Kemp, 2016); a recipient host of the same genus (*Chaetoceros* sp. KBDT32); and a more distantly related recipient, a pennate diatom (*Amphiprora* sp. KBDT35). Individual strains of *Alteromonas* and *Marinobacter* obtained from *Chaetoceros* sp. KBDT20 were inoculated into host diatom cultures to evaluate their effects on a host’s growth trajectory. The bacterial genera *Alteromonas* and *Marinobacter* are commonly associated with diatoms in culture (Amin, Parker & Armbrust, 2012). Genomic information suggests that both genera can have mutualistic interactions with a diatom host, based on their potential to provide B-vitamins including vitamin B_12_, however, this ability has not been confirmed for all species (Stahl & Ullrich, 2016; Garcia et al., 2017). Parasitism and commensalism have been documented as well. Some strains of *Alteromonas* have algicidal interactions with diatoms (Nelson & Wear, 2014; Wear et al., 2015) and some have been found to digest host setae (Cottrell et al., 2000) or inhibit host growth (Kim et al., 1999). Algicidal effects have been found to be highly host specific (Paul & Pohnert, 2011), which could lead to an ecological advantage for unaffected hosts and thereby influence the structure of diatom communities. Similarly, *Marinobacter* may be commensal (Gärdes et al., 2011; Gärdes et al., 2012; Stahl & Ullrich, 2016). Based on these previous reports, host responses to bacterial inocula could range from negative to neutral to positive impacts.

Previous studies suggest that bacteria-derived micronutrients, such as vitamins can be a key factor affecting diatom hosts. Therefore, this study also evaluates whether the impact of bacterial inoculations is modulated by the concentration of vitamins in the culture. The experiments were repeated in vitamin-replete and vitamin-deficient media in a fully-factorial, replicated experimental design. This experimental design allows us to evaluate the following possibilities: that bacterial inoculations may affect the growth trajectory of diatoms with an existing microbiome; that the origin of the bacterial inoculum (i.e., co-cultured vs. introduced to the host) may also influence the diatom growth trajectory; that congeneric diatom hosts may respond similarly to bacterial inoculations; that congeneric bacteria may induce similar responses in different diatom species; and that exogenous vitamins may affect the observed interactions between inoculated bacteria and a diatom host with an existing microbiome.

## Materials and Methods

### Experimental design

The experimental design is summarized in Table 1 and is explained in more detail in the following sections.

### Diatom culture collection

The diatoms evaluated in this study included the origin host from which the bacterial isolates were obtained (*Chaetoceros* sp. KBDT20), and two non-origin recipient hosts (*Chaetoceros* sp. KBDT32 and *Amphiprora* sp. KBDT35), hereafter referred to as non-origin hosts. The non-origin hosts had not been intentionally exposed to bacteria isolated from *Chaetoceros* sp. KBDT20, although related strains may exist in the non-origin host cultures (see below). All three diatom hosts were collected and identified by Christopher Schvarcz on May 8, 2011 from surface seawater in Kāneóhe Bay (O‘ahu, Hawai‘i) at the dock of the Hawai‘i Institute for Marine Biology. 18S rDNA was amplified as is described in DeLong (1992) and sequences are available at KU867951.1, MN422263, and MN422264. To initiate each diatom culture, a single founder cell and its attached microbiome were transferred into sterile seawater. Sterile seawater was prepared from Station ALOHA (22°45′N, 158°00′W) surface seawater filtered using a 0.1 µM Supor membrane AcroPak filter cartridge (Pall Corporation, Port Washington, NY, USA), and autoclaved for 30 min per L at 260 °C. The single-cell inoculum was grown in sterile seawater amended with the f/2 medium kit (NCMA, Boothbay, ME, USA), hereafter referred to as “vitamin-replete media”. Seawater amended with f/2 media prepared without vitamins will be referred to as “vitamin-deficient media”. The estimated vitamin concentrations in both media are discussed below. Cultures were grown at 15 µmol photons m^−2^sec^−1^ and 26 °C at a 12-hour light/12-hour dark cycle. Every 30–50 days, 0.5 mL of the culture was transferred to a 120 mL polycarbonate bottle with 50 mL of vitamin-replete media. Cultures were maintained following this procedure for three years prior to this study. Thus, these long-term cultures are the descendants of a single founder diatom cell and all co-cultured descendants of its original microbiome that persisted under these conditions.

**Table 1:** Experimental design to evaluate the impact of bacterial inoculations and the concentration of vitamins on the growth, carrying capacity, and decline of the host diatoms.

**Members and treatment**	
****	
**Host**	*Chaetoceros* sp. KBDT20 (origin)
1–10 cells µL^−1^	*Chaetoceros* sp. KBDT32 (non-origin)
	*Amphiprora* sp. KBDT20 (non-origin)
	
**Bacterial inoculum:**	*Alteromonas* 2016
50–100 cells µL^−1^	*Alteromonas* 2024
	*Alteromonas* scs5
	*Marinobacter*scs77
	*Marinobacter*scs85
	no-inoculum control
	
**Vitamin Treatment:**	Vitamin-replete media
	Vitamin-deficient media
	
**Experimental design**	
**Setup:**	Combine one from each: host, bacterial inoculum, and vitamin treatment


	Grown in 50 mL volume
	
**Replication:**	3 replicates of 36 combinations

**Sample frequency:**	48–72 h for 20–28 days
	

### Diversity of bacteria present in origin and non-origin diatom cultures

We examined the diversity of the existing microbiomes to provide context for the addition of single bacterial strains. Bacteria co-cultured with *Chaetoceros* sp. KBDT20 were described previously by Baker, Alegado & Kemp (2016). For the present study, bacteria co-cultured with *Chaetoceros* sp. KBDT32 and *Amphiprora* sp. KBDT35 were identified using 16S rDNA. Diatoms were grown to exponential phase, then homogenized using autoclaved 0.1 mm Zirconia/Silica beads (Biospec, Bartlesville, OK, USA) and centrifuged for 10 min at high speed at 4 °C. DNA from pelleted cells were extracted using the DNeasy Blood and Tissue Kit (Qiagen, Hilden, Germany), and the V4 region was amplified (Caporaso et al., 2012). Amplifications were cleaned and normalized using the SequalPrep™ Normalization Plate Kit (TheromoFisher Scientific, Waltham, MA, USA) and sequenced using pair-ended MiSeq (Illumina, San Diego, CA, USA) (PRJNA563321). Sequences were aligned and classified using Silva Incremental Aligner (SINA) in Silva (Pruesse, Peplies & Glöckner, 2012). Only sequences with greater than two occurrences in the sample were presented in the comparison of bacteria associated with the different diatom hosts. Additionally, because bacteria from *Chaetoceros* sp. KBDT20 were identified previously using a longer 16S rDNA region and Sanger sequencing, as compared to the V4 region presented here, sequences were only identified to order. Using the Basic Local Alignment Search Tool (BLAST), the subset of sequences identified as Alteromonadales associated with the non-origin hosts were aligned to *Marinobacter* and *Alteromonas* sequences that dominate *Chaetoceros* KBDT20. Sequences with 99% or greater similiarity to *Alteromonas* and *Marinobacter* from the Baker & Kemp (2014) study were submitted to GenBank (PRJNA382430).

### Isolation and identification of bacteria from the origin host

We isolated *Marinobacter* and *Alteromonas* similar to phylotypes previously found to dominate the *Chaetoceros* sp. KBDT20 microbiome using two methods described below; isolation methods are not intended as an experimental factor. The first method involved spreading 1 µL of diatom culture onto marine agar (Difco™ Marine Agar 2216, BD Biosciences, East Rutherford, NJ, USA) and incubating at 26 °C in the dark for 24 h. Ninety-six bacterial colonies were isolated using the T-streak method repeated in triplicate. Isolated colonies were grown in Difco™ marine broth 2216 (BD Biosciences, East Rutherford, NJ, USA) at 26 °C for 24 h before being preserved in glycerol (final concentration 30%) and stored at −80 °C. This approach resulted in isolates predominantly from a single genus. These sequences did not include *Marinobacter* phylotypes found in *Chaetoceros* sp. KBDT20 (Baker, Alegado & Kemp, 2016), so individual diatom cells were sorted using the BD Influx™ flow cytometer (BD Biosciences, East Rutherford, NJ, USA), using 0.2 µm filtered and autoclaved surface seawater from Station ALOHA as sheath fluid. The influx sheath tank was UVC sterilized for 12 h before adding sheath fluid and for two hours after adding sheath fluid. Cell sorting was performed at 6.0 psi sheath fluid pressure and 5.5 psi sample pressure using a 150 µM nozzle. Samples were triggered on 488-RED at a level of 26 and cells were gated using forward scatter (10^0.5^–10^2.5^) and 488-RED (10^2^–10^3.5^). The effectiveness of single-cell sorting of diatom cells was visually confirmed by microscopy before sorting into a 96-well culture plate containing marine agar. Contamination of the flow cytometer with any bacterial phylotypes that would grow on marine agar was assessed by sorting sterile seawater containing fluorescent beads into an additional 96-well culture plate. Plates were incubated in the dark at 26 °C and evaluated every 24 h for 5 days; negative controls were checked after an additional 11 days and were found to not contain any visible bacterial growth. From plates containing diatom cells, bacterial colonies were selected from twenty wells based on variations in colony morphology and time until first colony formation. Selected colonies were T-streaked on marine agar. Two to ten colonies of diverse colony morphology were selected from each of twenty T-streaked colonies, and these isolates were purified using three iterations of the streak plate method as described above. Bacterial isolates were confirmed to include a single phylotype by 16S rDNA sequencing. Bacterial DNA was isolated from colonies cultured in marine broth for 24 h. 1 µL of culture was diluted in 50 mL of sterile PCR clean water (Qiagen^®^, Hilden, Germany) before lysing using one cycle of freeze-thaw. Bacterial 16S rDNA was sequenced and evaluated as described in Baker, Alegado & Kemp (2016).

The 16S rDNA of bacterial cultures confirmed to contain a single strain were compared to the most prevalent *Alteromonas* and *Marinobacter* phylotypes in *Chaetoceros* sp. KBDT20 (Baker, Alegado & Kemp, 2016) (Supplemental Information 1). Five strains with 99% similar 16S rDNA to dominant bacteria from Baker, Alegado & Kemp (2016) were selected for further investigation and their sequences were submitted to GenBank (KY921850–KY921854). Strains were named based on the isolation method: 20XX isolates were obtained using the plating method, and scsXX isolates using the single cell FACS method. Similarity to strains isolated by other researchers was evaluated using BLAST, excluding uncultured organisms (Altschul et al., 1990). *Alteromonas* and *Marinobacter* sequences from Baker, Alegado & Kemp (2016), the new bacterial isolates, and the top BLAST hits were aligned using Silva/SINA and a tree was constructed using PhyML 3.0 (Guindon et al., 2010) (Fig. S1).

### Preparation and evaluation of bacterial inoculum

In low nutrient marine environments, bacteria rarely grow exponentially and are most often found in stationary phase (Kolter, Siegele & Tormo, 1993), during which bacteria may alter their metabolic pathways and express stress-response genes (Finkel, 2006). However, all bacterial isolates required elevated nutrient concentrations to grow to adequate concentrations for inoculations (data not shown). Bacterial cultures were grown to stationary phase in marine broth, followed by transfer to vitamin-deficient media for 24 h prior to inoculation into the host cultures. The transfer was to avoid contamination of the vitamin-deficient media with marine broth and to encourage the expression of possible stress-response genes characteristic of low-nutrient environments.

Marine broth was also used to evaluate bacterial growth in high nutrient conditions. Prior to inoculation, marine broth was filtered (0.2 µM polycarbonate filter, Corning Inc.) to remove particulates that would interfere with flow cytometric counts. Samples for bacterial cell counts were collected and preserved with 3% formaldehyde every 2–10 h, stained with SYBR^®^ (Thermo Fisher Scientific) to a concentration of 1X, and counted using an Attune flow cytometer (Thermo Fisher Scientific). The growth, maximum abundance, and decline of bacteria were determined in the same manner as described below for diatom cultures and were compared for bacterial genera and strains using lsmeans and one-way ANOVA, using the Companion to Applied Regression (car) package in R (Fox et al., 2015; Lenth, 2016). Any differences in the time required for different genera or strains to reach maximum abundance were noted.

To characterize how different bacterial strains respond to a low nutrient environment with no host present, bacteria grown for 48 h in marine broth were pelleted for 10 min at RCF 7,500× g at 4 °C using a Sorvall Primo R centrifuge (Thermo Fisher Scientific). The supernatant was removed, and the pellet was resuspended in vitamin-deficient media; this procedure was repeated twice to remove residual marine broth. Subsamples were plated on marine agar to confirm the survival of bacteria. Bacterial cell counts were taken as described above both after isolation and after 24 h of incubation in vitamin-deficient media.

### Bacterial and diatom culture preparation

Bacteria and diatoms were prepared separately and counted using an Attune flow cytometer before inoculation. Bacterial inoculum was counted by staining with 1X SYBR^®^ with the threshold set on BL1 (Excitation 488nm, Emission filter 530/30nm) at 10,000, and a voltage of 3,300; bacteria were gated at BL1-A (10^4.5^–10^7.5^) and SSC-A (10^1^–10^4.5^). Diatoms were grown to mid-exponential phase and counted using a threshold on BL3 (Emission filter > 640 nm) at 10,000, and a voltage of 2,550; diatom cells were gated using BL3-H (10^5.5^–10^6.5^) and VL2-H (10^2.5^–10^4.5^) (Emission filter 512/25 nm). Diatom cultures were then diluted to a concentration of 1–10 diatom cells µL^−1^ and bacterial cultures were diluted to a concentration of 50–100 bacterial cells µL ^−1^in 120 mL polycarbonate bottles containing either 50 mL of vitamin-replete medium or vitamin-deficient medium. The concentration of bacteria used in this study is similar to those employed in studies using axenic diatoms (Amin et al., 2015). Negative controls received a volume of sterile medium equal to the average inoculation volume of the corresponding bacteria-containing medium to avoid discrepancies in inoculated vs. control volumes. Each treatment and control evaluated was prepared in triplicate and kept at the same light and temperature conditions as the diatom culture collection. Cultures were gently swirled before sampling. Initial counts were taken within 2 h after bacteria and diatoms were added to new media (either vitamin-deficient or vitamin-replete media). These counts were repeated 19-26 h after the initial inoculation and then every 48–72 h to follow host and free-living bacterial abundances over time.

### Measuring and analyzing the effect of vitamins and bacterial inoculations on host cells

The effects of vitamin concentrations and bacterial inoculations on diatom growth, maximum abundance, and decline were evaluated for each diatom host. Parallel analyses of co-cultured free-living bacteria were also conducted. Analyses were performed on natural log-transformed count data for each replicate and time point. Diatom maximum abundance was defined as the average of the three highest consecutive counts for each replicate. Growth and decline was modeled using an equation developed by Churchill and Usagi (Membré, Thurette & Catteau, 1997, Eqn 3, Churchill & Usagi, 1972) using non-linear least squares as implemented in R (nlr). The Churchill/Usagi equation is:

\begin{eqnarray*}ln(N)=((1/{K}_{1}){e}^{-\lambda 1\ast t}+(1/{K}_{2}){e}^{\lambda 2\ast t})^{-1} \end{eqnarray*}

**Figure 1:**
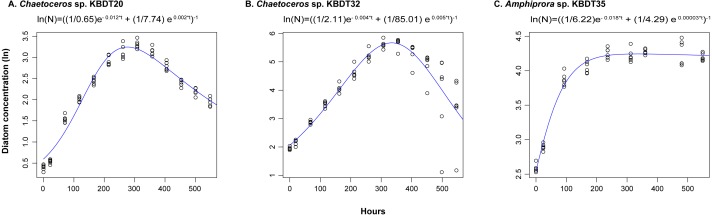
Flow cytometric counts of diatoms fit to the Churchill equation. Flow cytometric counts of diatoms from vitamin-replete experiments (black circles) fit to the Churchill equation (blue line) using non-linear least squares for (A) *Chaetoceros* sp. KBDT20, (B) Chaetoceros sp. KBDT32, and (C) Amphiprora sp. KBDT35. All three analysis were significant (*p* < 0.001) and the resulting equation for the set of points is given.

*λ*_1_ is a parameter describing growth during the log phase growth, hereafter referred to as growth exponent; K_1_ and K_2_ are constants that account for the initial density and peak density respectively; *λ*_2_ is the a parameter describing a log phase of decline after the culture reaches maximum abundance, hereafter referred to as the decline exponent; t is time in hours. Estimates of maximum abundance, exponential growth exponent (*λ*_1_), and decline exponent (*λ*_2_) were derived for each replicate. The data fit well to the diatom growth curves (Fig. 1); the fit of the data to each replicate is provided in Data S1.

The effects of (1) vitamin addition and (2) bacterial inoculation on the three growth parameters were evaluated for each diatom using linear modeling (lm) and estimated marginal means (emmeans) in R (R Development Core Team, 2011). Emmeans automatically adjusts for multiple comparisons and does not require additional post-hoc evaluation. Factors were considered significant if *p* < 0.05. The impact is calculated as the percent difference between the effector (vitamin-deficient conditions or bacterial inoculations) and the default conditions (i.e., vitamin-replete conditions or no bacterial inoculations, respectively) divided by the average of the two conditions. Negative impacts include decreased growth, lowered maximum abundance, or increased decline of the diatom host. Positive impacts include increased growth, increased maximum abundance, or decreased decline.

### Vitamin concentrations in vitamin-replete and vitamin-deficient media

Experiments were performed in both vitamin-replete and vitamin-deficient media. The source of sterilized surface seawater was from Station ALOHA cruise HOT 277 (October 12–16, 2015). The average concentrations of macronutrients for the month of October are well below the average diatom half saturation constant (K_s_, the nutrient concentration at which uptake is $ \frac{1}{2} $ of the optimal rate) (Table S1). The average vitamin concentrations at Station ALOHA are unknown. The current detection level for vitamin B_12_ is 29 pM (Sañudo Wilhelmy et al., 2012). An unpublished report on the vitamin concentrations at Station ALOHA in Fall 2012 documents B-vitamin concentrations at B_1_<20 pM, B_7_<5 pM, and B_12_<6 pM (D del Valle, pers. comm., 2012). The concentration of B_12_ at Station ALOHA may be near the average half saturation constant for diatoms (1.17 ± 0.77 pM), but autoclaving for over 30 min should result in degradation of B_12_ (Orrell & Caswell, 1972) to a resulting concentration lower than the half saturation constant. In vitamin-replete media, vitamins are added after f/2 media are autoclaved and brought to room temperature; the final concentrations are approximately 2.96 ×10^5^ pM vitamin B_1_, 2.05 ×10^3^ pM vitamin B_7_, and 3.69 ×10^8^ pM vitamin B_12_.

## Results and Discussion

**Figure 2:**
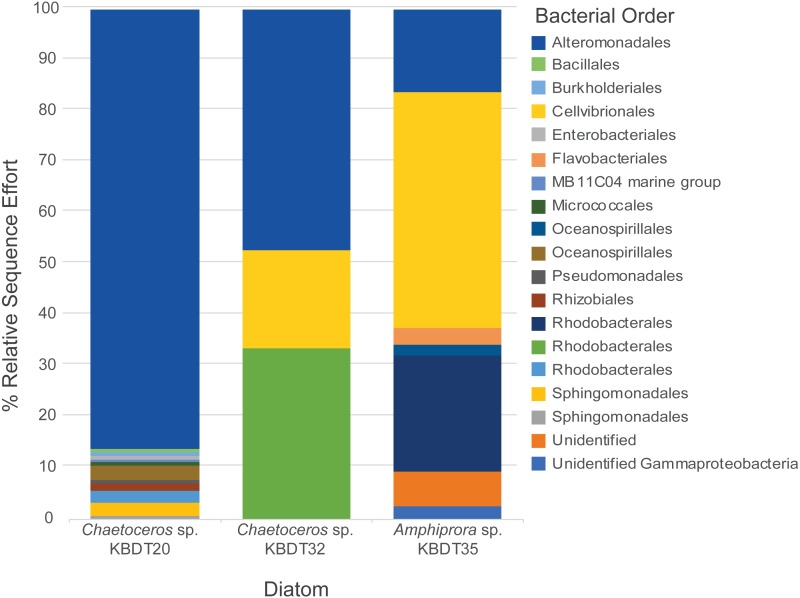
Bacterial orders associated with each diatom host. The bacteria associated with each diatom host, presented as the relative sequence effort, excluding all members with less than two sequences present in the effort. Due to a difference in sequencing technologies used to find *Chaetoceros* sp. KBDT20 16S rDNA sequences (longer Sanger sequencing reads), and *Chaetoceros* sp. KBDT32 and *Amphiprora* sp. KBDT35 (Illumina V4 sequencing), sequences were only identified to order.

This study expands on previous monoculture studies to more broadly examine the net effect bacterial inoculation has on a host with an existing bacterial consortium. Although each diatom evaluated in this study was collected from the same environment, each hosts a unique and complex microbiome (Fig. 2). However, because of the region amplified, these results may be overrepresenting members of the Gammaproteobacteria (Parada, Needham & Fuhrman, 2016). *Alteromonadales* was present in all xenic cultures and is the most prevalent order in both *Chaetoceros* cultures. Although all diatoms hosted similar (>99%) *Alteromonas* strains, neither non-origin culture hosted *Marinobacter* strains. Additionally, both non-origin strains host larger populations of *Cellvibrionales* (>20%) as well as seven bacterial orders that were not found during extensive sequencing of the origin culture *Chaetoceros* sp. KBDT20 (Baker, Alegado & Kemp, 2016). *Chaetoceros* sp. KBDT32 and *Amphiprora* sp. KBDT35 differed in their microbiomes, hosting one and five orders respectively unique to that diatom. This study was not designed to examine the influence of microbiome complexity, however, microbiome complexity plausibly could indirectly or directly influence the impacts of bacterial inoculations and/or vitamin concentrations on diatom cultures.

The bacterial inocula were isolated from the same host, *Chaetoceros* sp. KBDT20, but they significantly differed in their performance in culture, even between congeneric strains under optimal culture conditions (filtered, vitamin-replete marine broth at 26 °C) (Supplemental Information 2). Congeners *Alteromonas* 2016 and *Alteromonas* 2024 were 99% similar to *Alteromonas* scs5 using 16S rDNA, but the former two strains consistently formed aggregates and the latter did not (data not shown). The two bacterial genera also differed in their growth trajectory in optimal culture conditions; *Marinobacter* strains reached higher peak abundances but declined faster and maintained lower concentrations at stationary phase than *Alteromonas* strains. *Alteromona* 2016 grew faster and *Marinobacter* scs77 took the longest to reach maximum abundance relative to all other strains (Fig. S2). Similar to work by Amin, Parker & Armbrust (2012), all strains declined in vitamin-deficient media, suggesting that without an additional nutrient source such as a diatom host, all of these strains would be unable to grow in a vitamin-deficient medium.

**Figure 3:**
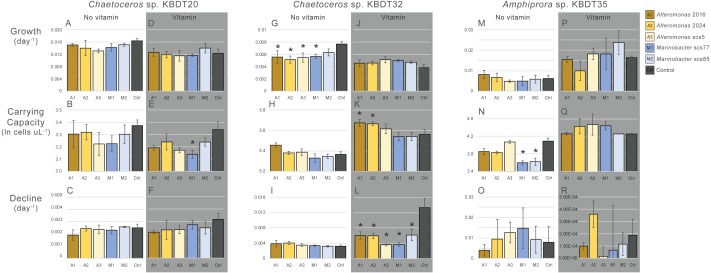
Modeled growth and decline and carrying capacity for each diatom and vitamin concentration. The average carrying capacity as well as growth and decline exponent from the Churchill equation of each diatom strain in vitamin deficient media (no vitamin) and vitamin replete media (vitamin). *Chaetoceros* sp. KBDT20 in no vitamin (A–C) and vitamin (D–F); *Chaetoceros sp.* KBDT32 in no vitamin (G–I) and vitamin (J–L); and *Amphiprora* sp. KBDT35 in novitamin (M–O) and vitamin (P–R). Significant impacts of inoculation with *Alteromonas* (shades of yellow) or *Marinobacter* (shades of blue) relative to the control (shaded in dark grey) are denoted with an asterisk. Error is shown as standard deviation.

The concentration of bacterial inoculum used in this study was comparable to that used in previous studies of axenic diatoms (Amin et al., 2015); the initial diatom culture contained approximately 50–100 bacterial cells per µL^−1^ (Fig. S3 at time 0). At these concentrations, the bacterial inoculum had very little impact on the growth trajectory of the origin host but had more frequent significant impacts on the non-origin hosts (Fig. 3, significant impacts are listed in Table 2). It is not surprising that only minor impacts were observed for *Chaetoceros* sp. KBDT20; it has been in co-culture with its associated bacteria for years, and the number of bacterial cells in an inoculum represent only a slight perturbation to its microbiome.

**Table 2:** Significant impacts of bacterial inoculations in (A) vitamin-deficient and (B) vitamin- replete conditions relative to the control. Non-significant values are not included. The percentage impact is given. Beneficial impacts, such as the percentage increase in growth or carrying capacity and decrease in the decline, are not highlighted. Detrimental impacts, such as the decrease in the growth exponent or carrying capacity and an increase in the decline exponent, are highlighted in grey.

**Diatom**	**Stage**	**Inoculation comparison**		**Impact (%)**	***p*-value**
**A. Vitamin-deficient**					
*Chaetoceros* sp. KBDT32	Growth	*Alteromonas* 2016	Control	−32	0.003
		*Alteromonas* 2024	Control	−40	<0.001
		*Alteromonas* scs5	Control	−34	0.002
		*Marinobacter*scs77	Control	−30	0.004
*Amphiprora* sp. KBDT35	Carrying capacity	*Marinobacter*scs77	Control	−13	<0.001
		*Marinobacter*scs85	Control	−12	<0.001
					
**B. Vitamin-replete**					
*Chaetoceros* sp. KBDT20	Carrying capacity	*Marinobacter*scs77	Control	−6	0.030
*Chaetoceros* sp. KBDT32	Carrying capacity	*Alteromonas* 2016	Control	2	0.016
		*Alteromonas* 2024	Control	2	0.035
	Decline	*Alteromonas* 2016	Control	−75	<0.001
		*Alteromonas* 2024	Control	−77	<0.001
		*Alteromonas* scs5	Control	−115	<0.001
		*Marinobacter*scs77	Control	−114	<0.001
		*Marinobacter*scs85	Control	−74	<0.001

Inoculation with bacteria isolated from a different host resulted in varying impacts to the growth trajectory of the non-origin hosts, *Amphiprora* sp. KBDT35 and *Chaetoceros* sp. KBDT32. We had expected that *Chaetoceros* sp. KBDT32 might respond similarly to *Chaetoceros* KBDT20, as they were in the same host genus. However, *Chaetoceros* sp. KBDT32 experienced the greatest number of significant impacts of all hosts tested, as well as the highest percentage impact to its growth trajectory particularly with regard to a reduction in its decline exponent. In contrast, the more distantly related *Amphiprora* sp. KBDT35 experienced only 12 and 13% impacts to its growth trajectory, specifically on carrying capacity (Table 2). In this experiment, the impact of added bacteria was not predicted by host relatedness.

Because the origin culture was minimally impacted by bacterial inoculations, it can be postulated that bacterial inoculations into a culture that already hosts similar strains should have little to no impact. By extension, introduction of bacteria not already present in a xenic diatom culture may be more likely to cause a significant impact on the diatom’s growth trajectory. Inoculation with either strain of *Marinobacter* did impact the growth trajectory of both non-origin strains, neither of which had *Marinobacter* in their existing microbiomes. Results were mixed for inoculation of non-origin hosts with *Alteromonas* strains. Both non-origin diatoms hosted *Alteromonas* that were 99% similar to those hosted by *Chaetoceros* sp. KBDT20. Although *Amphiprora* sp. KBDT35 is not impacted by inoculation with *Alteromonas* strains, all growth parameters of *Chaetoceros* sp. KBDT32 were impacted by *Alteromonas* (although the impact varies by nutrient condition, discussed below). Therefore, the presence of similar bacteria is an inconsistent predictor of impact to these strains.

Overall, inoculation with *Marinobacter* and *Alteromonas* did not have a consistently positive or negative impact on any of the diatom hosts. Several studies have documented harmful impacts of *Alteromonas* on diatoms, but commensal interactions with *Marinobacter* (Kim et al., 1999; Cottrell et al., 2000; Bidle & Azam, 2001; Paul & Pohnert, 2011; Gärdes et al., 2011; Gärdes et al., 2012; Stahl & Ullrich, 2016). In the present study, inoculation with *Alteromonas* often resulted in a small but significant positive impact on maximum abundance of the diatom host, relative to inoculation with *Marinobacter* (Table 3). However, these significant differences were small and occurred too sporadically to conclude that there is a generalizable difference between the effects of *Alteromonas* vs. *Marinobacter* (i.e., neither genus has a consistently more positive nor more negative net effect on hosts).

**Table 3:** Significant comparisons between the impact of bacterial inoculations, where the percentage impact compares the inoculum in the first column to the inoculum in the second column, in both vitamin-deficient (A) and vitamin-replete (B) media. Highlights indicate when the first inoculum is detrimental relative to the second. In the remaining comparisons, the first inoculum was beneficial relative to the second.

**Diatom**	**Stage**	**Inoculation comparison**		**Impact (%)**	***p*-value**
**A. Vitamin-deficient**					
*Chaetoceros* sp. KBDT32	Carrying capacity	*Alteromonas* 2016	*Marinobacter*scs77	2	0.005
		*Alteromonas* 2016	*Marinobacter*scs85	2	0.023
Amphiprora sp. KBDT35	Carrying capacity	*Alteromonas* scs5	*Marinobacter*scs77	12	<0.001
		*Alteromonas* scs5	*Marinobacter*scs85	12	<0.001
**B. Vitamin-replete**					
*Chaetoceros* sp. KBDT32	Carrying capacity	*Alteromonas* 2016	*Marinobacter*scs77	2	0.002
		*Alteromonas* 2016	*Marinobacter*scs85	2	0.003
		*Alteromonas* 2024	*Marinobacter*scs77	2	0.006
		*Alteromonas* 2024	*Marinobacter*scs85	2	0.006
*Amphiprora* sp. KBDT35	Growth	*Alteromonas* 2024	*Marinobacter*scs85	−82	0.003

Inoculation with different bacterial strains from the same genus rarely had identical impacts on the trajectory of a diatom host. No significant differences were found between strains from the same genus (Table 3). However, in many instances inoculation with one strain of bacteria significantly impacted the growth, maximum abundance, or decline of a diatom host, while inoculations of congeneric strains had no effect (Table 2). When more than one congeneric bacterial strain significantly affected the trajectory of a host, the magnitude of the effect varied greatly among strains (1–40%). Some previous studies using axenic diatoms also found that bacterial strains with nearly identical 16S-rDNA can impact the host differently (Sison-Mangus et al., 2014; Zecher et al., 2015). Phylogeny based on 16S rDNA appears to be a poor predictor of strain-specific effects of bacteria on xenic diatoms.

We anticipated that one or more of the bacterial strains could provide B-vitamins that were absent from vitamin-deficient media (Garcia et al., 2017). If true, we would expect bacterial inoculations in vitamin-deficient media to be beneficial to the diatom, while inoculations in vitamin-replete media would not. Instead, we observed nearly the opposite (Table 2); most significant effects in vitamin-deficient media were negative, and most significant effects in vitamin-replete media were positive. Clearly, our results do not support a conclusion that these bacterial strains provided vitamins to the host diatoms. Our results are further complicated by finding that higher vitamin concentration had a negative impact on the growth of the controls for both *Chaetoceros* cultures (Table 4). In these xenic cultures, the effect could be a result of indirect interactions; for example, an abundance of vitamins could favor the growth of bacteria harmful to the diatoms and the lack of vitamins could be negated by hosting a microbiome capable of producing sufficient concentrations of B-vitamins.

**Table 4:** Significant percentage impact of vitamin concentrations for a given inoculation state (i.e., bacterial inocula or control). The percentage impact compares vitamin-replete conditions to vitamin deficient conditions; i.e., (replete deficient)/(replete + deficient). Non-significant values are not included. Cases where added vitamins are beneficial include an increase in growth exponent or carrying capacity, or a decrease in the decline exponent (not highlighted). Cases where added vitamins are detrimental include a decrease in the growth exponent or carrying capacity, or an increase in the decline exponent (highlighted in grey).

**Diatom**	**Stage**	**Inoculation**	**Impact (%)**	***p*-value**
*Chaetoceros* sp. KBDT20	Growth	Control	−28	0.026
*Chaetoceros* sp. KBDT32	Growth	Control	−63	<0.001
		*Marinobacter*scs85	−29	0.038
	Carrying capacity	*Alteromonas* 2016	4	<0.001
		*Alteromonas* 2024	5	<0.001
		*Alteromonas* scs5	4	<0.001
		*Marinobacter*scs77	4	<0.001
		*Marinobacter*scs85	4	<0.001
		Control	4	<0.001
	Decline	Control	122	<0.001
		*Marinobacter*scs85	63	0.036
*Amphiprora* sp. KBDT35	Growth	*Alteromonas* scs5	116	0.004
		*Marinobacter*scs77	115	0.004
		*Marinobacter*scs85	121	<0.001
	Carrying capacity	*Alteromonas* 2016	10	0.001
		*Alteromonas* 2024	15	0.000
		Alteromonasscs5	9	0.002
		*Marinobacter*scs77	4	0.000
		*Marinobacter*scs85	21	0.000

Although the impact of bacterial inoculations on the origin host *Chaetoceros* sp. KBDT20 was not impacted by vitamin concentrations, the impact of inoculations on non-origin hosts was modulated by vitamin concentration (Table 2). For non-origin host *Chaetoceros* sp. KBDT32, bacterial inoculations significantly suppressed the host growth exponent in vitamin-deficient conditions (i.e., a negative impact) but increased its carrying capacity and decreased its decline exponent (i.e., positive impacts) in vitamin-replete conditions. It is possible that the effect of bacterial inoculations may depend on whether the host is under stress from other causes such as vitamin deficiency. Negative impacts to the carrying capacity of *Amphiprora* sp. KBDT35 were observed in vitamin-deficient media, but positive impacts were not observed in vitamin-replete conditions. These results suggest that the net effects of introducing bacteria from another host can be impacted by vitamin concentrations, but the interaction is not universal.

## Conclusions

The number of possible influencing factors is obviously greater in experiments employing a xenic model system, compared to axenic systems. However, we have shown that it is possible to address meaningful questions in xenic systems, and we would argue that xenic systems are likely to be more realistic in ecological and algaculture contexts. This study suggests that perturbations of the diatom-associated bacterial microbiome may profoundly impact fundamental properties of non-origin diatom populations such as growth, maximum abundance, and decline. Additionally, closely related bacterial strains can have very different impacts even on closely related diatom hosts. The responses of non-origin hosts were influenced by the concentration of vitamins. This suggests that when xenic diatoms are exposed to unfamiliar strains of bacteria, their interaction can be influenced by abiotic stressors, such as vitamin concentration. Our findings advocate for further investigation of diatom-bacterial interactions in diatom cultures with existing microbiomes, exploring the resistance and resilience of the host’s microbiome when perturbed, and recognizing that the environmental context (e.g., vitamins) may be important. The existence of complex host-bacterial and microbiome-bacterial interactions may be vital to the health, success, or failure of diatom species in nature.

##  Supplemental Information

10.7717/peerj.8352/supp-1 Supplemental Information 1 Supplemental methods, results, figures, and tables

10.7717/peerj.8352/supp-2 Supplemental Information 2 Rates of each parameter to evaluate the impact to host trajectory The growth rate, average peak, and rate of decline for each replicate. The diatom evaluated, bacterial inoculum, and vitamin treatment is listed.

10.7717/peerj.8352/supp-3 Supplemental Information 3 Growth and decline from the Churchill equation for each replicate The output of the Churchill equation (lambda1 or growth and lambda2 or decline) fit to various timepoints

10.7717/peerj.8352/supp-4 Supplemental Information 4 Chaetoceros KBDT20 growth curves modeled with Churchill/Usagi equation Flow cytometric count data for each replicate of Chaetoceros KBDT20 modeled using the Churchill/Usagi equation

10.7717/peerj.8352/supp-5 Supplemental Information 5 Growth curve of Chaetoceros KBDT32 replicates modeled with the Churchill/Usagi equation Flow cytometric count data for each replicate of Chaetoceros KBDT32 modeled using the Churchill/Usagi equation

10.7717/peerj.8352/supp-6 Supplemental Information 6 Growth curve of Amphiprora replicates modeled using the Churchill/Usagi equation Flow cytometric count data for each replicate of Amphiprora sp. KBDT35 modeled using the Churchill/Usagi equation

10.7717/peerj.8352/supp-7 Supplemental Information 7 Raw sequence data awaiting approval from NCBI, PRJNA563321
Raw sequence for bacteria cocultured with Amphiprora sp. KBDT35 data awaiting approval from NCBI, PRJNA563321

10.7717/peerj.8352/supp-8 Supplemental Information 8 Raw sequence data awaiting approval from NCBI, PRJNA563321
Raw sequence data of bacteria cocultured with Chaetoceros KBDT32 awaiting approval from NCBI, PRJNA563321

## Additional Information and Declarations

### DNA Deposition

The following information was supplied regarding the deposition of DNA sequences: 16S sequences for the bacterial inoculum are available at GenBank (KY921850–KY921854). The bacteria present in the non-origin cultures are available at GenBank: PRJNA382430; Chaetoceros sp. KBDT32: MN857903–MN859961; Amphiprora sp. KBDT35: MN857753–MN857893.

### Data Availability

The following information was supplied regarding data availability: Data are available as Supplemental Files.
