# Supplementary material for: Bacterial inoculations can perturb the growth trajectory of diatoms with an existing microbiome
Source: PeerJ. 2020 Jan 27;8:e8352. doi: 10.7717/peerj.8352 (PMC6991125; doi:10.7717/peerj.8352)
Supplement: Supplemental Information 1 [file peerj-08-8352-s001.doc]

**Supplemental Material**

*I. Diversity of bacteria isolated from* Chaetoceros *sp. KBDT20*

A total of 65 sequences from the direct plating method and 61 sequences from the single-cell isolation method were found to be single-phylotype amplifications. The direct plating isolates were predominantly *Alteromonas* (93%); all other isolates were identified as *Erythrobacteracea* (7%). In contrast, only 30% of bacteria isolated by FACS were *Alteromonas*; the majority of bacteria (70%) were identified as *Marinobacter*. Sequences from both methods clustered at 99% similarity using mothur (Schloss *et al.*, 2009) resulted in 14 distinct strains: 7 *Alteromonas*, 6 *Marinobacter*, and 1 *Erythrobacter*.

Bacteria were selected for further analysis based on their similarity to prevalent strains identified in past experiments using *Chaetoceros* sp. KBDT20 (Supplemental Fig. 1). *Marinobacter* strains scs77 and scs85 were similar (99% in BLAST) to strains observed in our previous work (strain KM283562; Baker et al. 2016), as well as to *Marinobacter* isolated from sponges (Esteves *et al.*, 2016), and to *Marinobacter* sp. SA55 and *Marinobacter* sp. SA50 isolated from *Pseudonitzschia multiseries* (Amin *et al.*, 2015)*. Alteromonas* strains 2016, 2024, and scs5 also were selected. *Alteromonas* scs5 was closely related to the most prevalent strain isolated in our previous work (strain KM382524; Baker et al. 2016). The remaining *Alteromonas* strains all were closely related (99% similar using mothur), and similar to amplified *Alteromonas* strains observed in our previous work (strain KM383636; Baker et al. 2016). They were also closely related (99% identical in BLAST) to strains that were found to influence the settling of the coral *Pocillopora damicornis* (Tran and Hadfield, 2011) or found in association with the sea sponge *Rhabdastrella globostellata* (Steinert *et al.*, 2014). *Alteromonas* 2016 and 2024 were chosen based on their unique growth physiology in culture; after 24 hours in filtered marine broth, *Alteromonas* 2016 and 2024 formed aggregates but neither formed biofilms on glass or plastic culture vessels. All other bacterial isolates utilized in this study grew evenly suspended in marine broth.

*II. Bacterial inoculum growth, carrying capacity, and decline*

*Marinobacter* and *Alteromonas* strains had similar growth rates in marine broth, but differed in their carrying capacity, rate of decline, and final concentration at stationary phase (Supplemental Fig. 2). The mean growth rate for all strains was 2.89±0.15 day^-1^ and did not vary significantly among genera (p=0.589). *Marinobacter* had a significantly higher carrying capacity than *Alteromonas* (9%; p= 0.0006); *Marinobacter* had a carrying capacity of 2.62±0.18 x 10^4^ cells μL^-1^ and *Alteromonas* had a carrying capacity of 2.31±0.05 x 10^4^ cells μL^-1^. *Marinobacter* scs77 required 24 hours to reach carrying capacity, while all other bacteria reached carrying capacity within 18 hours of inoculation. *Marinobacter* declined significantly faster than *Alteromonas* (p<0.0001), and all bacteria were at stationary phase after 42 hours of growth. At stationary phase, *Marinobacter* persisted at significantly lower concentrations (48% lower) than *Alteromonas* (p=0.0003). Individual strains acted as described for genera, with the exception of *Alteromonas* scs5. *Alteromonas* scs5 had a significantly faster growth rate (44% faster; p=0.006), but did not significantly differ from other *Alteromonas* or *Marinobacter* strains in carrying capacity, decline, or concentration at stationary phase.

*III. Growth of free-living bacteria in some diatom co-cultures effected by host and vitamin concentrations*

Because xenic diatom cultures contain both attached and free-living bacteria, some co-cultured bacteria were transferred when diatom cultures are diluted into new media. This high background of free-living bacteria in the diatom cultures, coupled with the error associated with our counting method, prevented an accurate count of the bacterial inoculum at the time of the first free-living bacterial count. The estimated concentration of free-living bacteria transferred with the diatom host at the start of the experiment was 79 ± 21 cells μL^-1^ transferred with *Chaetoceros* sp. KBDT20 cultures, 48 ± 12 cells μL^‑1^ transferred with *Chaetoceros* sp. KBDT32 cultures, and 100 ± 29 cells μL^-1^ transferred with *Amphiprora* sp. KBDT35 cultures. Bacterial inoculum was diluted to add 50-100 bacteria per μL^-1^ to the xenic culture; however, the between-replicate variation in counts of free-living bacteria was similar to the concentration of the bacterial inoculum, precluding measurements of the initial bacterial inoculum (data not shown). Inoculations had no discernable effect on the maximum concentration of free-living bacteria in each culture, compared to negative controls (p>0.05).

*Supplemental Table 1:* Nutrient concentrations for marine diatoms under varying nutrient regimes. The range of observed half saturation constants (Ks) for marine diatoms (Sarthou et al. 2005) for nitrogen, phosphorous, and silica. The Ks for vitamin B_12_ is based on studies of cultured algae (Droop 2007). The half saturation constant is the nutrient concentration at which uptake is ½ of the optimal rate. Concentration ranges for Station ALOHA were average and standard errors taken at the surface (from 5-25 m) from 1988-2015. Nutrient concentrations of f/2 are taken from Guillard and Ryther 1962. All values listed are in µM.

| *Nutrient* | *Average Ks* | *n* | *Range Concentration Station ALOHA* | *Concentration in f/2 media* |
| --- | --- | --- | --- | --- |
| *Nitrogen* | 1.6 ± 1.9 | 35 | (3.16 ± 0.64) x 10^-3^ | 882 |
| *Phosphorous* | 0.24 ± 0.29 | 18 | (46.29 ± 8.60) x 10^-3^ | 36.2 |
| *Silica* | 3.9 ± 5.0 | 25 | 1.17 ± 0.05 | 106 |
| *Vitamin B_12_* | (1.17 ± 0.77) x 10^-6^ | 13 | <6 x 10^-6^ | 3.69 x 10^-4^ |

*Supplemental Fig. S1*: A tree of the bacterial strains selected for further analysis (labeled with +) compared to *Alteromonas* and *Marinobacter* phylotypes from Baker et al., 2016 (labeled with *, with *** being the most prevalent strain), as well as the top five BLAST hits for each strain; there was some overlap for each genus. For each sequence downloaded from BLAST, the ID, genus and species name, and the isolation source is listed for each if available.


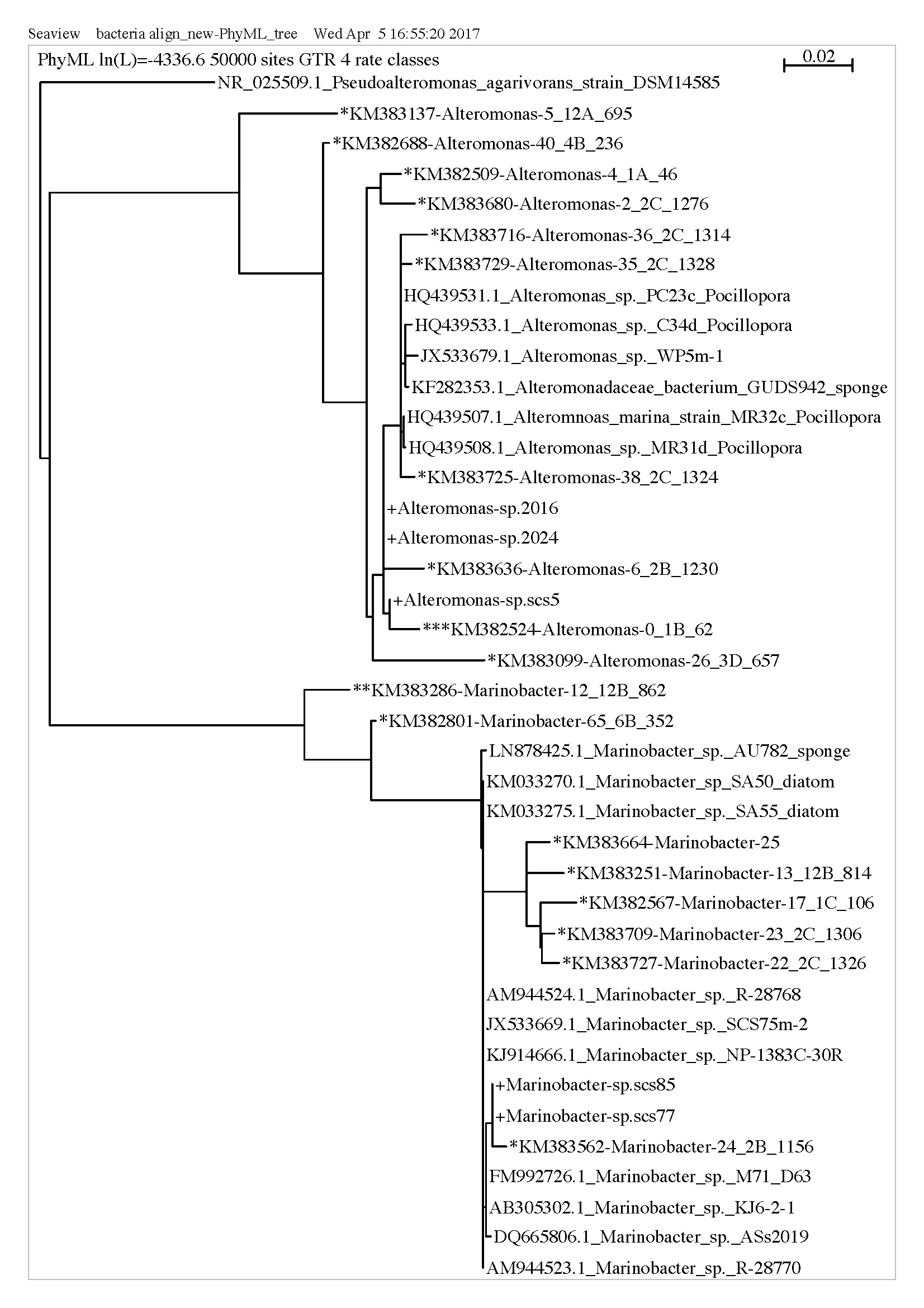


*Supplemental* *Fig. S2*: Cell counts of bacterial isolates used to determine the rate of growth and decline. This was performed for bacteria grown in marine broth (0-48 h) and in vitamin deficient f/2 (48-72 h). Transfer to new f/2 vitamin-deficient media at 48 hours denoted by arrow. Error shown is standard error.

*Supplemental Fig. S3*: Growth of free-living bacteria (square and x) that is both cocultured and assumedly some inoculum, measured at the same timepoint as and diatoms (circle and cross) in vitamin deficient media (no vitamin) and vitamin replete media (vitamin). The presence of bacterial inoculum (black lines) and without bacterial inoculum (grey lines) are also listed. Error is shown as standard error.


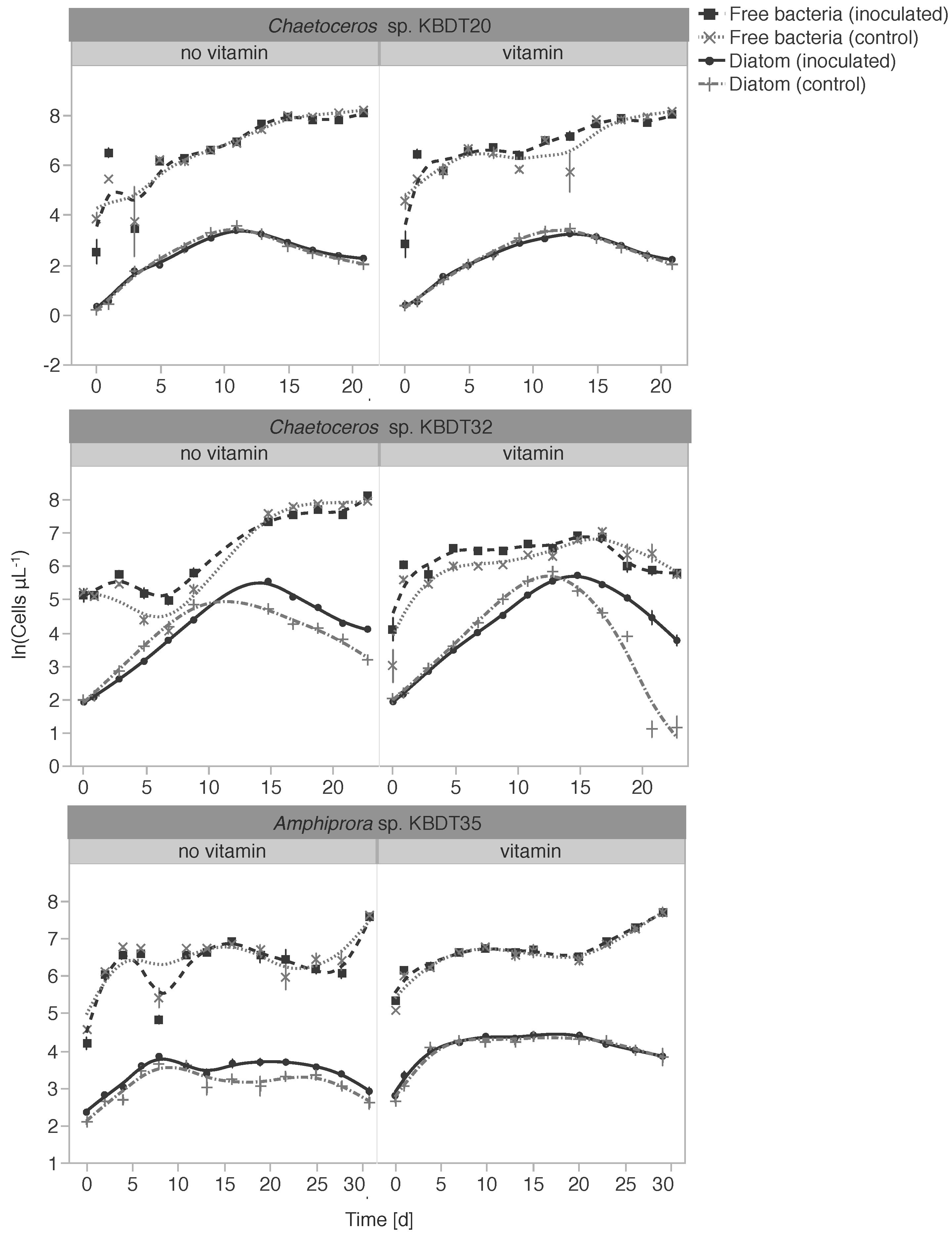


**References**

Amin, S.A., Hmelo, L.R., van Tol, H.M., Durham, B.P., Carlson, L.T., Heal, K.R., et al. (2015) Interaction and signalling between a cosmopolitan phytoplankton and associated bacteria. *Nature* **522**: 98–101.

Baker, L.J.L.J.L.J., Alegado, R.A.R.A., and Kemp, P.F.P.F. (2016) Response of diatom-associated bacteria to host growth state, nutrient concentrations, and viral host infection in a model system. *Environ. Microbiol. Rep.*

Esteves, A.I., Amer, N., Nguyen, M., and Thomas, T. (2016) Sample processing impacts the viability and cultivability of the sponge microbiome. *Front. Microbiol.* **7**: 1–17.

Guillard RRL, Ryther JH. 1962. Studies of marine planktonic diatoms. I. *Cyclotella nana* Hustedt and *Detonula confervacea* Cleve. Canadian Journal of Microbiology 8:229§239

Schloss, P.D., Westcott, S.L., Ryabin, T., Hall, J.R., Hartmann, M., Hollister, E.B., et al. (2009) Introducing mothur: open-source, platform-independent, community-supported software for describing and comparing microbial communities. *Appl. Environ. Microbiol.* **75**: 7537–41.

Steinert, G., Whitfield, S., Taylor, M.W., Thoms, C., and Schupp, P.J. (2014) Application of Diffusion Growth Chambers for the Cultivation of Marine Sponge-Associated Bacteria. *Mar. Biotechnol.* **16**: 594–603.

Tran, C. and Hadfield, M.G. (2011) Larvae of Pocillopora damicornis (Anthozoa) settle and metamorphose in response to surface-biofilm bacteria. *Mar. Ecol. Prog. Ser.* **433**: 85–96.
