# Supplementary figures and images for: Bacterial inoculations can perturb the growth trajectory of diatoms with an existing microbiome
Source: PeerJ. 2020 Jan 27;8:e8352. doi: 10.7717/peerj.8352 (PMC6991125; doi:10.7717/peerj.8352)

**Chaetoceros sp KBDT32**

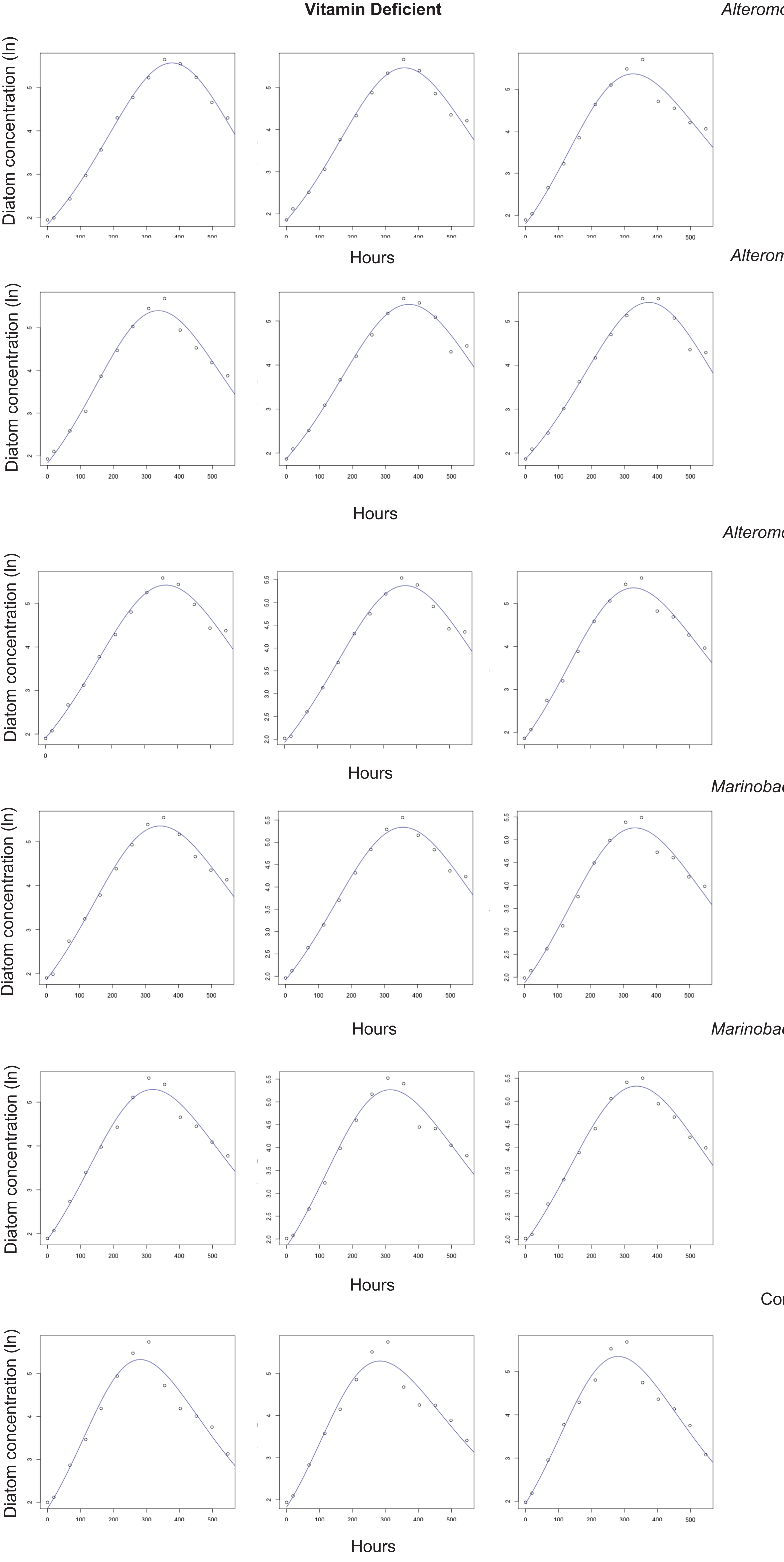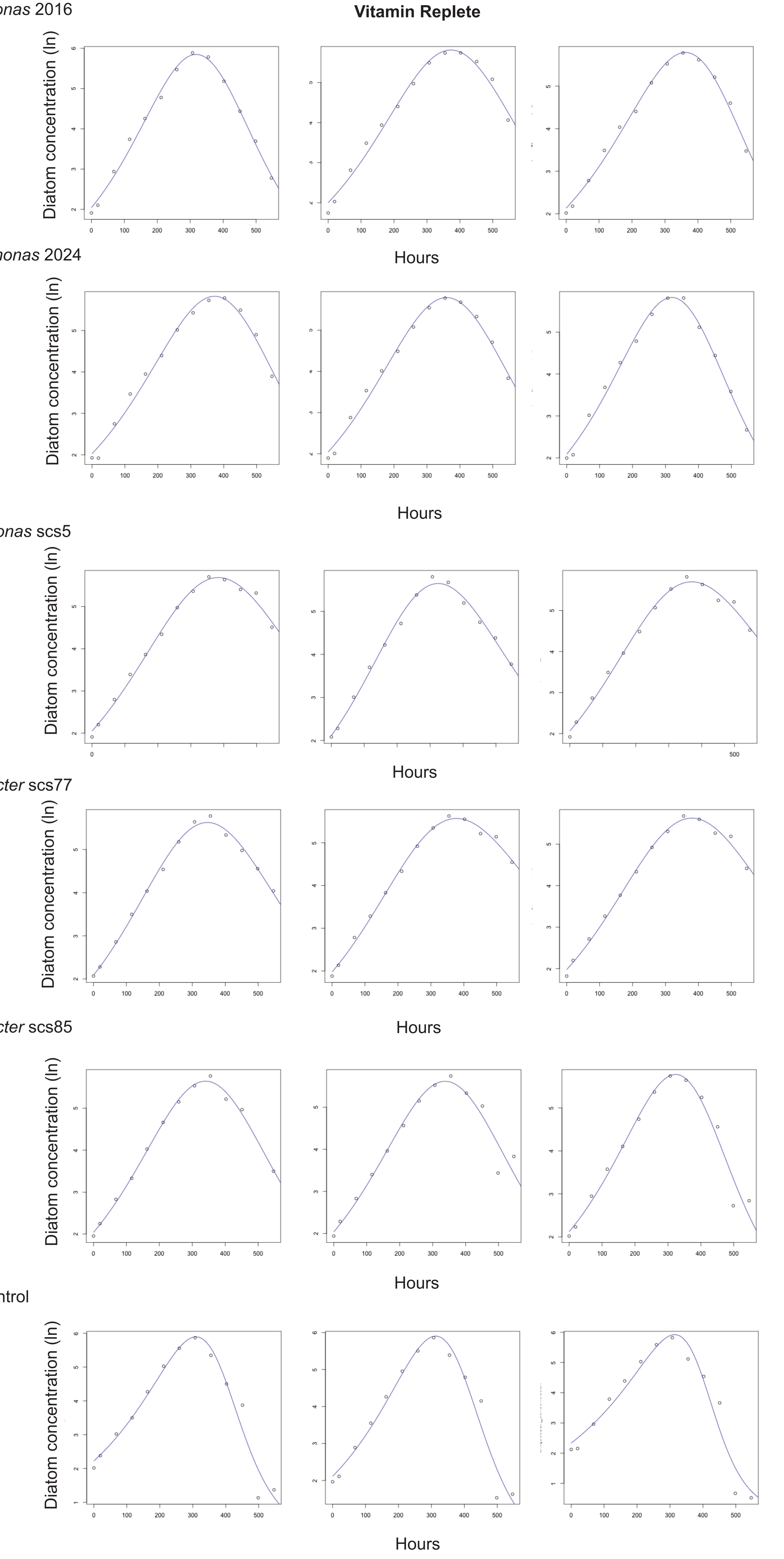

Supplement: Supplemental Information 5 — Flow cytometric count data for each replicate of Chaetoceros KBDT32 modeled using the Churchill/Usagi equation [file peerj-08-8352-s005.pdf]
