# Supplementary material for: Bacterial inoculations can perturb the growth trajectory of diatoms with an existing microbiome
Source: PeerJ. 2020 Jan 27;8:e8352. doi: 10.7717/peerj.8352 (PMC6991125; doi:10.7717/peerj.8352)

***Amphiprora* sp. KBDT35**

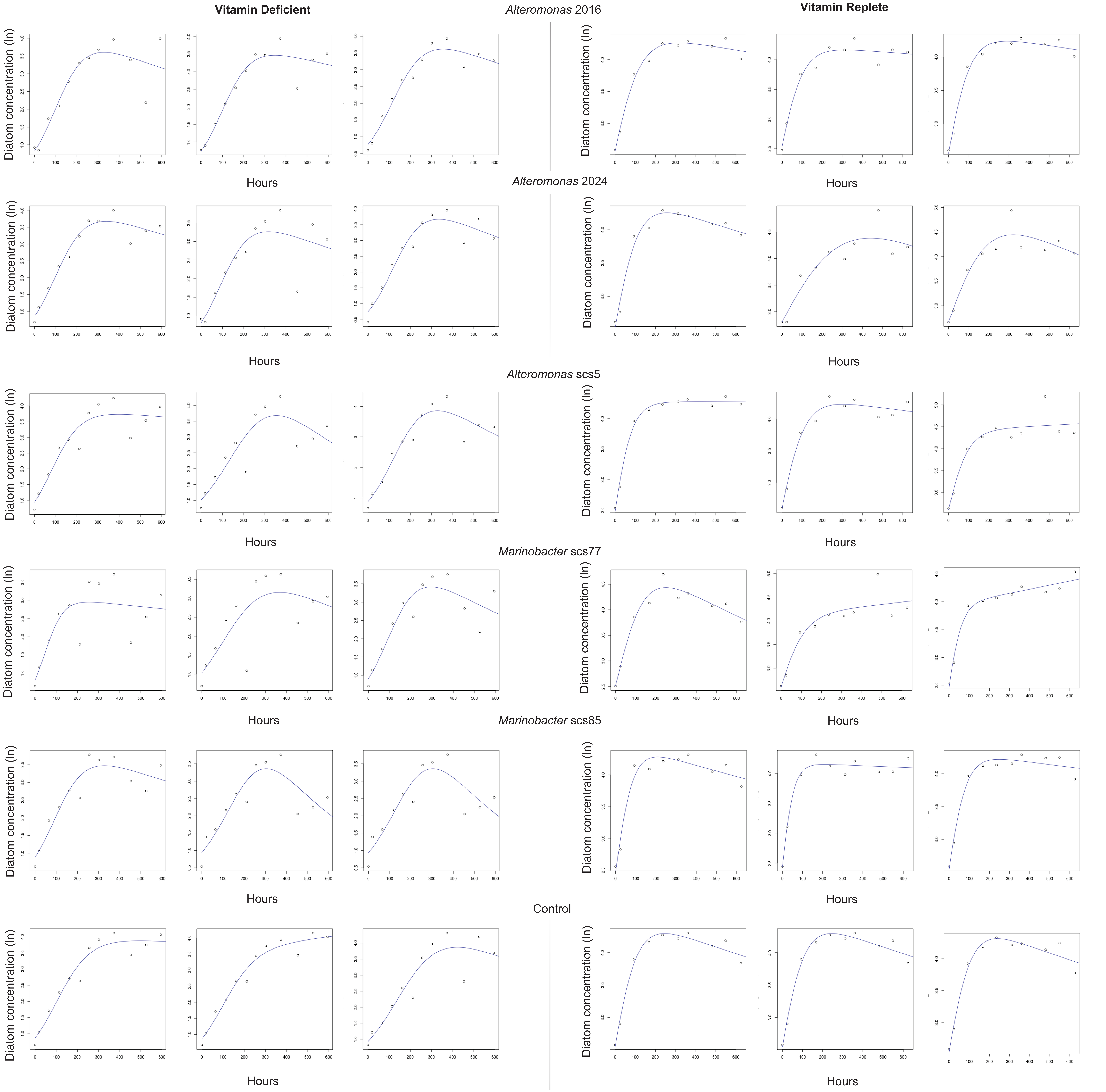

Supplement: Supplemental Information 6 — Flow cytometric count data for each replicate of Amphiprora sp. KBDT35 modeled using the Churchill/Usagi equation [file peerj-08-8352-s006.pdf]
