# Supplementary material for: Bacterial inoculations can perturb the growth trajectory of diatoms with an existing microbiome
Source: PeerJ. 2020 Jan 27;8:e8352. doi: 10.7717/peerj.8352 (PMC6991125; doi:10.7717/peerj.8352)
Supplement: Supplemental Information 4 — Flow cytometric count data for each replicate of Chaetoceros KBDT20 modeled using the Churchill/Usagi equation [file peerj-08-8352-s004.pdf]

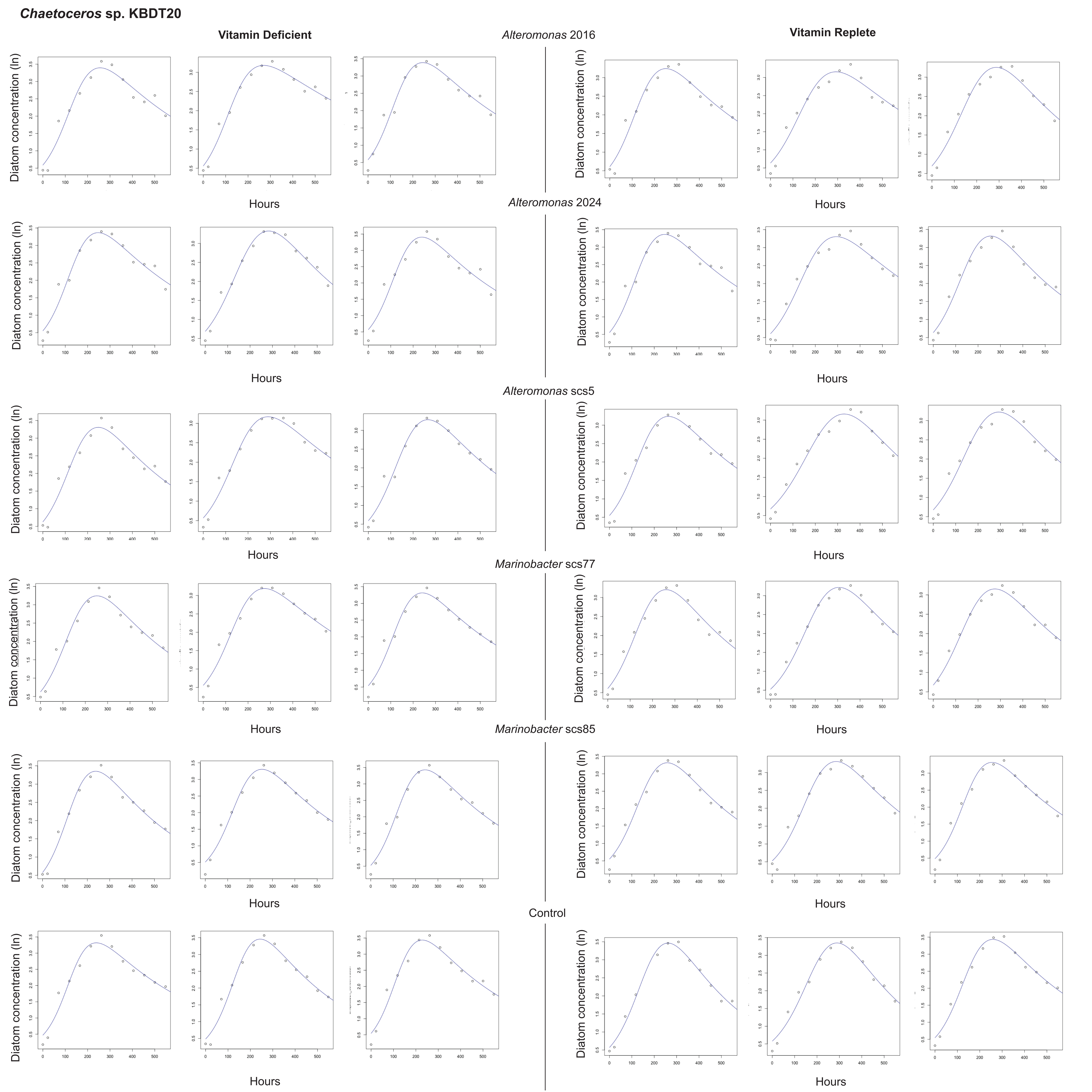
Chaetoceros sp. KBDT20
Vitamin Deficient
Vitamin Replete
Alteromonas 2016
Alteromonas 2024
Alteromonas scs5
Marinobacter scs77
Marinobacter scs85
Control
Diatom concentration (ln)
Hours
